# Post-encoding task engagement not attentional load is detrimental to awake consolidation

**DOI:** 10.1038/s41598-024-53393-6

**Published:** 2024-02-06

**Authors:** Michael Craig, Joanna Greer

**Affiliations:** Department of Psychology, Faculty of Health and Life Sciences, Northumbria University, Newcastle Upon Tyne, UK

**Keywords:** Human behaviour, Consolidation

## Abstract

The fate of new memories depends partly on the cognitive state experienced immediately following encoding. Wakeful rest, relative to task engagement, benefits retention and this effect is typically explained through a consolidation account: rest is theorised to provide a state of minimal interference, which would otherwise disrupt consolidation. Yet, the determinants of consolidation interference, notably the contribution of attention, remain poorly characterised. Through a repeated measures design, we investigated attention load’s impact on consolidation. In three phases, participants encountered a set of nonwords and underwent immediate recognition testing, experienced a 5-min delay condition, and completed a delayed recognition test for the nonwords. This cycle repeated for each phase before proceeding to the next. Delay conditions comprised of wakeful rest and two sustained attention to response tasks (SART) that were of low (SART-fixed) and high (SART-random) attention load. Immediate memory was matched across conditions, but delayed recognition was poorer after completing the SART-fixed and SART-random conditions, relative to rest. There was no difference between the two SART conditions. These data provide insights into the factors that contribute to the success of consolidation and indicate that the attention load of a task does not determine the magnitude of consolidation interference and associated forgetting.

## Introduction

Consolidation refers to the process through which new labile memories are strengthened and stabilised over time^1,2^. This process is theorised to be opportunistic and occurs predominantly in the absence of task engagement, including during quiescent states of sleep and wakeful rest^3–5^. Behavioural evidence supports this: even a brief period of wakeful rest in the immediate aftermath of encoding benefits the retention of verbal, visual, and spatial information^6–16^. These effects of rest, which cannot be explained by mnemonic strategies^11^, are not transient but durable over a time course of days to weeks^9,17^. Wakeful rest is proposed to benefit the retention of new memories because it provides a state of minimal interference—through the absence of an engaging task—that would otherwise disrupt mechanisms of consolidation, for example, the neural reactivation of novel memory traces ^6,16,17^. Indeed, the neural reactivation of recently encoded traces is found to occur especially during quiescent states like wakeful rest, and the magnitude of reactivation in the immediate post-encoding period positively predicts memory retention^18–20^.

Contributions of quiescence and task engagement to wakeful consolidation is an emerging topic and has largely been examined through studies focusing their investigations around (i) how these two states affect the retention of verbal, visual, and spatial information^6,9,15,16,21–25^, (ii) whether states of wakeful rest and task engagement affect consolidation differently in cohorts varying in age and memory capacity^10,26–30^, and (iii) revealing the neural underpinnings of wakeful consolidation^12,31–33^. Despite valuable contributions from these inquiries, consolidation in the awake state remains poorly characterised, including the factors that determine its success and an individual’s susceptibility to consolidation interference from task engagements.

Still, some insights surrounding these gaps in the current literature can be drawn from existing data. Engagement with a task in the immediate aftermath of encoding is detrimental to consolidation when these activities comprise spot-the-difference games^6,9,11,17,23,25^, tone detection and listening tasks^16^, visual search tasks^34^, abstract visual problems^7,8^, video games^12,24^, and vivid autobiographical thinking in response to auditory cues^13,34^. Common to these various activities is the delivery or retrieval of sensory information that is often rich in episodic context. This could hint towards sensory load being a driving force behind consolidation interference, possibly because the processing and retrieval of sensory codes induces concurrent memory activity that disrupts ongoing consolidation^6,17^. Some evidence for this possibility exists; while episodically rich stimuli reliably produce interference effects, findings are mixed when paradigms utilise stimuli that are not as rich in their episodic properties. For example, studies have failed to observe an interference effect, relative to wakeful rest, through the application of auditory cues that are devoid of episodic context^34^, numeric stimuli in the form of an n-back task^35^ and Raven’s advanced progressive matrices (visual problem solving) that are traditionally used to probe abstract reasoning^8^.

Whilst it is plausible that sensory load may contribute to consolidation interference, a further common factor across tasks that have induced an interference effect is sustained attention to incoming sensations or internal mentation^7,8,12,21,22,25,34^. It is therefore possible that—in addition to sensory load—the attention load associated with task engagement may contribute to observed effects in memory retention. This possibility resonates with neurobiological theories and evidence suggesting that states of alertness, including periods of task engagement, provide a neurophysiological state that is conducive to the encoding of new memories but less so to consolidation, whereas the opposite is true during quiescent task-free states like wakeful rest^4,5^.

Behavioural data provide further support for this possibility. Individual differences in working memory, which is closely associated with attentional control^36–38^, have been found to moderate consolidation interference effects, where superior working memory is associated with greater interference from task engagement^39^. This finding was postulated to reflect superior working memory ability resulting in a more focused state of attention, which was detrimental to consolidation. Furthermore, the extent of attention directed internally towards meditative breathing techniques has been found to positively predict the magnitude of consolidation interference observed for visually presented words^40^. Specifically, participants who successfully focused on their breathing for the majority of a 10-min retention interval demonstrated greater forgetting of the words compared to those who focused on their breathing for less than half of the delay condition. It is also intriguing that the comparison of tasks requiring internally and externally directed attention indicates that one is not more detrimental than the other, such tasks produce comparable interference, possibly because they induce comparable states of sustained attention^34^.

These findings suggest that a sustained state of attention may be detrimental to consolidation. It is worth noting that some contrasting evidence exists. When manipulating the demand of an n-back task, no interference effect, relative to rest, has been observed^35^. Such findings may be accounted for by employed tasks not being sufficiently demanding in attention to induce an interference effect, or with studies being underpowered to detect subtle effects in memory retention^24^. Nevertheless, there is value in characterising the determinants of consolidation interference associated with task engagement, including identifying the possible contribution of attention load when controlling for factors such as sensory load.

To contribute towards this, through an online experiment, the current study investigated whether consolidation interference can be explained, at least partly, by the attention load of an engaging task completed in the minutes immediately following encoding. A total of 120 younger adults (18–35 years old), recruited through Prolific, encoded three lists of nonwords and completed an immediate and delayed recognition test for the stimuli before and after three 5-min delay conditions. Delay conditions comprised of eyes-closed rest and two sustained attention to response tasks (SART) that were of low (SART-fixed) or high (SART-random) attention load. Crucially, in using two variations of the SART, attentional load could be manipulated while maintaining a consistent sensory load across tasks. Based on existing evidence and theories, we hypothesised that (i) completion of an engaging task should be detrimental to consolidation, relative to a period of quiet rest, and (ii) should the attention load of an engaging task influence the magnitude of consolidation interference, a task of greater attentional load should result in poorer memory – measured as increased forgetting – than an equivalent task of lesser attentional load.

## Results

Three participants were removed from the recruited sample of n = 120 because they were more than three standard deviations from the sample mean in their memory performance (n = 2) or did not sufficiently comply with the requirements of one or more aspects of the task procedure (n = 1). Thus, the following analyses report data from a final sample of n = 117. Still, no results changed when these excluded participants were retained in our sample for analyses. 

### Demographics

The mean age of the final sample was 28.22 years (SD = 4.24). A total of 55 participants (/117, 47.00%) identified as men (including trans man), 57 (/117, 48.70%) identified as women (including trans woman), 3 (/117, 2.60%) identified as non-binary, and 2 (/117, 1.70%) self-identified their gender as agender (n = 1) and gender fluid (n = 1). When probed on their highest level of educational attainment, 4 participants (/117, 3.40%) reported O levels/GCSEs or below, 39 (/117, 33.30%) reported College or Sixth Form A levels, 43 (/117, 36.80%) reported an Undergraduate degree, 22 (/117, 18.80%) reported a Postgraduate degree, and 9 (/117, 7.70%) reported holding a professional or Doctoral degree (e.g., PhD or MD). 

### Memory performance

#### Immediate recognition

**Figure 1:**
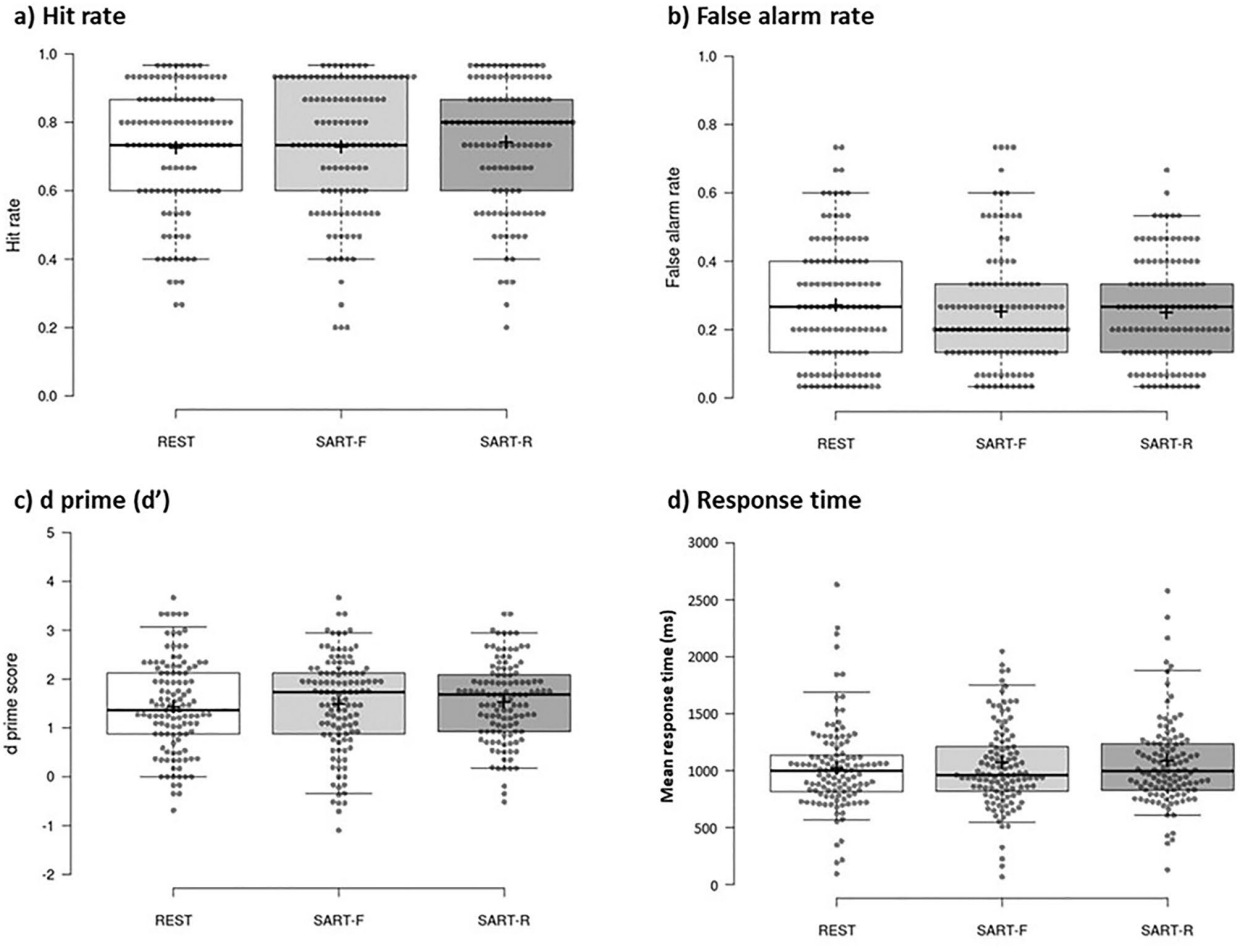
Immediate recognition test performance. The box plot shows immediate recognition test (**a**) hit rates, (**b**) false alarm rates, (**c**) d’ primes, and (**d**) mean response times (ms) for the rest (white), SART-fixed (light grey), and SART-random (dark grey) delay conditions. Centre lines show the medians; box limits indicate the 25th and 75th percentiles; whiskers extend to the 5th and 95th percentiles; crosses represent sample means; data points are plotted as open circles. Hit rates, false alarm rates, d’ prime scores, and mean response times were comparable across the three delay conditions.

Figure 1 reports hit rates, false alarm rates, d’ scores, and response times for the three delay conditions in the immediate recognition test. In this test, the three delay conditions were matched in their hit rates (rest: mean = 0.73, SD = 0.18; SART-F: mean = 0.73, SD = 0.20; SART-R: mean = 0.74, SD = 0.18; F(2,232) = 0.391, P = 0.677, η_p_^2^ = 0.003), false alarm rates (rest: mean = 0.27, SD = 0.18; SART-F: mean = 0.25, SD = 0.17; SART-R: mean = 0.25, SD = 0.15; F(2,232) = 0.911, P = 0.403, η_p_^2^ = 0.006), and d’ scores (rest: mean = 1.45, SD = 0.95; SART-F: mean = 1.50, SD = 0.97; SART-R: mean = 1.54, SD = 0.81; F(2,232) = 0.663, P = 0.532, η_p_^2^ = 0.005).

No effect of delay condition was observed in overall response times (ms) in the immediate recognition test (rest: mean = 1030 ms, SD = 380 ms; SART-F: 1080 ms, SD = 620 ms; SART-R: mean = 1090 ms, SD = 510 ms; F(2,232) = 0.949, P = 0.388, η_p_^2^ = 0.006). Similarly, when breaking test stimuli down, there was no main effect of delay condition in responses to old (target) items (rest: mean = 1000 ms, SD = 420 ms; SART-F: mean = 1030 ms, SD = 570 ms; SART-R: mean = 1050 ms, SD = 490 ms; F(2,232) = 0.532, P = 0.588, η_p_^2^ = 0.005) or new (foil) items (rest: mean = 1050 ms, SD = 400 ms; SART-F: mean = 1110 ms, SD = 710 ms; SART-R: mean = 1130 ms, SD = 580 ms; F(2,232) = 1.137, P = 0.322, η_p_^2^ = 0.010). For hit rates, false alarm rates, d’ scores, and response times, paired t-tests (two-tailed) confirmed no significant differences in pairwise comparisons (all P ≥ 0.190).

Comparable immediate recognition test performance across the three conditions indicates that any differences observed in delayed recognition test performance, probed after our experimental manipulation, is unlikely to be explained by differences in the initial encoding and retention of nonwords. 

#### Delayed recognition

**Figure 2:**
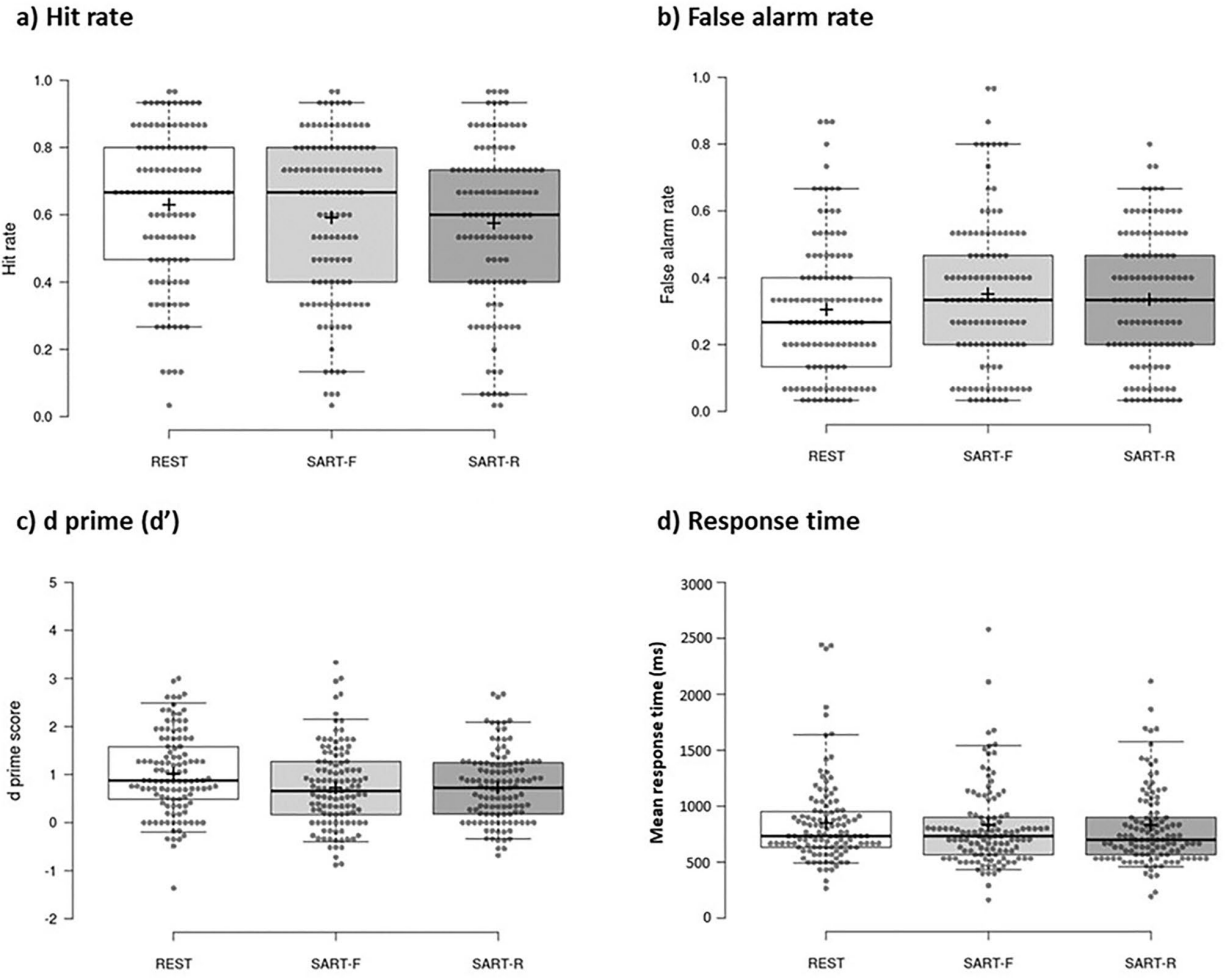
Delayed recognition test performance. The box plot shows delayed recognition test (**a**) hit rates, (**b**) false alarm rates, (**c**) d’ primes, and (**d**) mean response times (ms) for the rest (white), SART-fixed (light grey), and SART-random (dark grey) delay conditions. Centre lines show the medians; box limits indicate the 25th and 75th percentiles; whiskers extend to the 5th and 95th percentiles; crosses represent sample means; data points are plotted as open circles. While hit rates, false alarm rates, and mean response times to targets were comparable across the three delay conditions, d’ prime scores were superior in the rest delay condition than in the SART-fixed and SART-random conditions.

Figure 2 reports hit rates, false alarm rates, d’ scores, and response times for the three delay conditions in the delayed recognition test, which probed a different set of stimuli to the immediate recognition test. Analysis of delayed recognition test data revealed no significant main effect of delay condition in hit rates (rest: mean = 0.63, SD = 0.22; SART-F: mean = 0.59, SD = 0.24; SART-R: mean = 0.58, SD = 0.24; F(2,232) = 2.090, P = 0.126, η_p_^2^ = 0.018) or false alarm rates (rest: mean = 0.31, SD = 0.21; SART-F: mean = 0.35, SD = 0.22; SART-R: mean = 0.34, SD = 0.20; F(2,232) = 2.120, P = 0.122, η_p_^2^ = 0.016). Planned pairwise comparisons (two-tailed) revealed that hit rates were significantly greater following rest than the SART-R condition (t(116) = −2.144, P = 0.034, d = −0.198) but this did not survive a Bonferroni-corrected alpha level of 0.017 (P = 0.050/3 comparisons). All other tests were non-significant (all P ≥ 0.055).

A significant effect of delay condition was observed in d’ scores (rest: mean = 1.01, SD = 0.84; SART-F: mean = 0.74, SD = 0.85; SART-R: mean = 0.74, SD = 0.73; F(2,232) = 6.725, P = 0.001, η_p_^2^ = 0.055). Paired t-tests (two-tailed) revealed significantly greater d’ scores following rest compared to both the SART-F condition (t(116) = −2.998, P = 0.003, d = −0.277) and SART-R condition (t(116) =  −3.438, P < 0.001, d = −0.503). Both findings survived a Bonferroni-corrected alpha level of 0.017 (P = 0.050/3 comparisons). No significant difference emerged between the SART-F and SART-R conditions (t(116) =  −0.023, P = 0.982, d = −0.002). To further explore this null finding in delayed recognition test d’ scores between the SART-F and SART-R conditions, a Bayesian paired samples t-test was conducted. This test provided *strong* (BF_01_ ≥ 10) evidence^41^ in favour of the null hypothesis (BF_01_ = 13.558), i.e., that there was no difference in delayed d’ scores between the SART-F and SART-R conditions. 

Response time data from the delayed recognition test revealed no main effect of delay condition in the duration to respond (ms) across all trials (rest: mean = 860 ms, SD = 390 ms; SART-F: mean = 830 ms, SD = 450 ms; SART-R: 830 ms, SD = 510 ms; F(2,232) = 0.304, P = 0.738, η_p_^2^ = 0.003), old (target) items only (rest: mean = 860 ms, SD = 440 ms; SART-F: mean = 790 ms, SD = 460 ms; SART-R: mean = 770 ms, SD = 540 ms; F(2,232) = 1.738, P = 0.178, η_p_^2^ = 0.015), and new (foil) items only (rest: mean = 880 ms, SD = 450 ms; SART-F: mean = 890 ms, SD = 550 ms; SART-R: mean = 910 ms, SD = 540 ms; F(2,232) = 0.276, P = 0.759, η_p_^2^ = 0.002). Paired t-tests (two-tailed) comparisons confirmed no significant differences in response time between all items, target items only, and foil items only (all P ≥ 0.336). A further three repeated measures ANOVAs with within-subject factors delay condition (3 levels) and time of test (2 levels) revealed significant main effects of time across response times to all items, targets only, and foils only (all P < 0.001), with participants faster to respond in the delayed recognition test in all cases.

To establish whether there was an effect of time on memory, three repeated measures ANOVAs with within-subject factors delay condition (three levels: Rest vs. SART-F vs. SART-R) and time of recognition test (two levels: immediate vs. delayed) revealed significant main effects of time across hit rate, false alarm rates, and d’ scores (all P < 0.001) with poorer performance in the delayed recognition test in all cases. Similarly, a repeated measures ANOVA with response time data revealed a significant effect of time (F(1,116) = 45.129, P < 0.001, η_p_^2^ = 0.280), with participants faster to respond in the delayed recognition test than in the immediate recognition test. There was no significant main effect of delay condition (F(2,232) = 0.159, P = 0.853, η_p_^2^ = 0.001) or interaction between time and delay condition (F(2,232) = 1.972, P = 0.853, η_p_^2^ = 0.001).

### Delay condition SART performance

**Figure 3:**
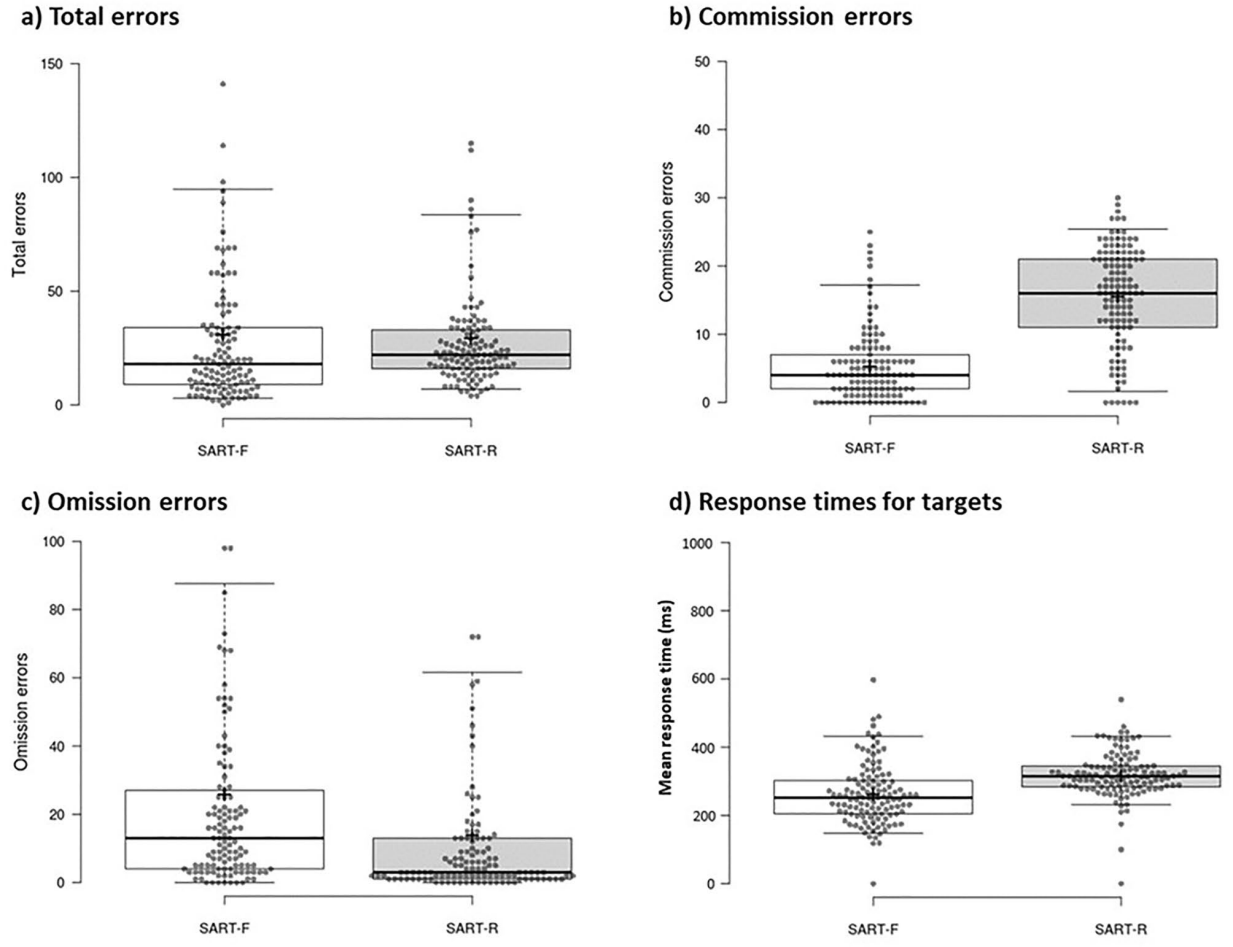
Performance in the sustained attention to response tasks. The box plot shows the mean number of (**a**) total errors, (**b**) commission errors, (**c**) omission errors, as well as (**d**) mean response times (ms) to target items for the SART-fixed (white) and SART-random (light grey) conditions. Centre lines show the medians; box limits indicate the 25th and 75th percentiles; whiskers extend to the 5th and 95th percentiles; crosses represent sample means; data points are plotted as open circles. The total number of overall errors was comparable between conditions though participants made significantly more omission errors in the SART-fixed condition and significantly more commission errors in the SART-random condition. Mean response times for target items were significantly slower in the SART-random condition.

Participants did not complete a task during the rest delay, where they were simply asked to sit quietly and relax with their eyes closed. During the remaining two delay conditions, participants completed a sustained attention to response task (SART)^42^. Two variations were used: the SART-F used a fixed order of stimuli presentation (digits 1 to 9 presented in a sequential order, i.e., 1–2-3–4-5–6-7–8-9–1–2…), and the SART-R used a random order of stimuli presentation (digits 1 to 9 presented in a random order, e.g., 4–5–7–6–3–1–2–8–9–2–6…). In both variations, participants responded to all items except the digit 3, where response inhibition was required. Omission errors refer to a missed response, i.e., where no response was provided to digits 1–2 and 4–9. Commission errors refer to failed inhibition, i.e., where a response was incorrectly made to the digit 3. Because of this structure, the SART-R is typically considered to place greater demand on attentional resources. Figure 3 shows participants’ performance in the SART-F and SART-R conditions.

Paired t-tests (two-tailed) revealed no significant difference in the total number of overall errors in the SART-F and SART-R tasks (SART-F: mean = 31.22, SD = 42.30; SART-R: mean = 29.71, SD = 30.20; t(116) = 0.385, P = 0.701, d = -0.036). However, participants did make a significantly greater number of commission errors (failed response inhibition) in the SART-R (SART-R: mean = 15.64, SD = 7.40) than in the SART-F (mean = 5.29, SD = 5.36) condition (t(116) = −13.153, P < 0.001, d = −1.216), which would be expected should the SART-R be more attentionally demanding. A significantly greater number of omission errors (failure to respond) were observed in the SART-F (mean = 25.93, SD = 41.92) than in the SART-R (mean = 14.07, SD = 30.92) condition (t(116) = 2.946, P = 0.004, d = 0.272). Participants were significantly slower to respond to targets (digits 1, 2, and 4–9), where a spacebar response was required, in the SART-R (mean = 330 ms, SD = 60 ms) than in the SART-F (mean = 260 ms, SD = 90 ms) condition (t(116) = −7.685, P < 0.001, d = −0.714), which, similar to a greater number of commission errors, is indicative of a greater attentional load in the SART-R task.

Because our study was delivered remotely and online, which meant we could not verify participants level of engagement or the determinants of performance variability, a conservative approach was adopted for the SART conditions, and no outlier thresholds were employed to the full dataset to minimise data attrition. However, it is worth noting that when a threshold of 3 standard deviations was applied, to remove extreme outliers, five participants were identified as meeting this criterion. Still, removal of these participants from our sample did not change any of our results.

### Delay condition mental activities

A total of 78 (/117, 66.70%) participants self-reported—as instructed—keeping their eyes closed for the duration of the 5-min rest delay condition, while 39 (/117, 33.30%) participants reported not keeping their eyes closed for the duration of this condition. When asked whether they employed any form of mnemonic strategy to help retain recently presented nonwords, 40 participants (/117, 34.20%) reported using some form of mnemonic strategy during the rest delay, whereas 19 participants (/117, 16.20%) reported utilising a strategy in the SART-F delay and 14 participants (/117, 12.00%) reported using a strategy in the SART-R delay. When participants who reported employing a strategy during the rest delay were removed from our sample, the earlier reported significant main effect in d' scores remained (rest: mean = 0.98, SD = 0.86; SART-F: mean = 0.70, SD = 0.91; SART-R: mean = 0.75, SD = 0.73; F(2,152) = 4.100, P = 0.018, η_p_^2^ = 0.051). Similarly, pairwise comparisons continued to demonstrate that delayed d’ scores were significantly greater in the rest condition than the SART-F (t(76) = −2.355, P = 0.021, d = −0.267) and SART-R (t(76) = −2.616, P = 0.011, d = −0.298) conditions, and there was still no significant difference between the SART-F and SART-R conditions (t(76) = −0.429, P = 0.669, d = −0.049).

A significant main effect of delay condition was found in ratings of the regularity of thoughts pertaining to the presented nonwords (rest: mean = 2.23, SD = 1.11; SART-F: mean = 1.68, SD = 0.95; SART-R: mean = 1.44, SD = 0.76; F(2,232) = 37.585, P < 0.001, η_p_^2^ = 0.246). A main effect of delay condition was also observed in the regularity that participants (i) imagined the nonwords (rest: mean = 2.06, SD = 1.05; SART-F: mean = 1.55, SD = 0.90; SART-R: mean = 1.43, SD = 0.80; F(2,232) = 28.660, P < 0.001, η_p_^2^ = 0.198) and (ii) consciously remembered the nonwords (rest: mean = 2.44, SD = 1.35; SART-F: mean = 1.69, SD = 1.02; SART-R: mean = 1.50, SD = 0.89; F(2,232) = 41.905, P < 0.001, η_p_^2^ = 0.265). Pairwise comparisons (two-tailed) revealed that, in all cases, the significant main effects of the delay condition were due to participants reporting significantly greater regularity of thoughts, imaginations, and remembering of nonwords during the rest delay than the SART-F delay (all P < 0.001) and SART-R conditions (all P < 0.001). No significant differences were observed between SART-F and SART-R conditions (all P ≥ 0.093). Irrespective of the noted differences between conditions, mean scores for each question demonstrate that the level of thoughts about the nonwords was modest across the three delay conditions, where a score of 1 corresponded to *“not at all”* and a score of 5 to *“constant”*. 

## Discussion

This study aimed to establish whether the attention load of an engaging task contributes to consolidation interference. In three phases, participants encountered a set of nonwords and underwent immediate recognition testing, experienced a 5-min delay condition, and completed a delayed recognition test for the nonwords. This cycle repeated for each phase before proceeding to the next. Delay conditions comprised eyes-closed rest and two sustained attention to response tasks (SART)^42^ that were of low (SART-fixed) or high (SART-random) attention load. Conditions were matched in immediate memory, but poorer memory was observed following both SART delays, relative to rest. No evidence for attention load determining consolidation interference was found; memory for the nonwords was comparable following the SART-fixed and SART-random conditions. We discuss these findings and possible explanations in turn. 

The observation of reduced memory following a period of task engagement, relative to rest, is in keeping with published work^6–16^. Counterbalancing of wordlists and delay conditions means that it is unlikely that this effect can be explained by noise from our methodological approach or differences in the encoding of wordlist stimuli. This is supported by our data: memory scores and response times were comparable in the immediate recognition test for the three conditions, which indicates that participants encoded wordlists sufficiently—and to a similar degree—in all conditions. Thus, the difference in *delayed* memory performance is unlikely to be explained by divergences in the initial encoding of stimuli. Additionally, because target items differed between the immediate and delayed tests, it is unlikely that the act of retrieving memories in the immediate recognition test contributed to the effect in the delayed recognition test, which is pertinent given that evidence indicates retrieval can act as a fast route to consolidation^43^. Comparable response times in the delayed recognition test also suggest that participants were able to retrieve stored memories to a similar level across delay conditions. This hints towards reduced memory following a filled delay being the result of differences in memory availability rather than accessibility.

Given that participants' immediate recognition test performance was comparable across the three delay conditions, a more probable explanation for reduced memory following completion of a sustained attention task, relative to rest, is that the activities during these filled and unfilled conditions affected encoded traces differently. One possibility is that participants demonstrated reduced forgetting following rest because this unfilled, task-free state provided an opportunity for mnemonic strategies including the retrieval of encoded stimuli, which is known to act as a fast route to consolidation^43^. Indeed, while most participants self-reported adhering to rest delay instructions and keeping their eyes closed for the duration of the delay, we found that a greater proportion of participants reported thoughts pertaining to the encoded stimuli during rest than task engagement. However, such thoughts are unlikely to fully explain the observed effect in memory because (i) nonword stimuli (e.g., *cartolale*) were chosen purposefully as they are challenging to retrieve and rehearse, (ii) effects of rest in memory have been found to not depend on intentional rehearsal^11^, (iii) even though participants reported greater frequency of thoughts on nonword stimuli during rest, the magnitude of these thoughts was relatively modest across all delay conditions and corresponded to “not much” to “sometimes”, and (iv) when participants who reported using a strategy during rest were excluded from our analyses, differences in d’ scores between the rest and two SART conditions remained significant. 

Instead, we propose that this effect can be explained through a consolidation account. Specifically, we postulate that rest provided a state that is conducive to the early consolidation of new memory traces^1,2^. This hypothesis is in keeping with existing literature demonstrating rest-related effects in the retention of new memories^6–16,23^. Rest is thought to benefit consolidation because it provides a state of minimal task engagement that would otherwise disrupt consolidation^6,16,17^. Indeed, in the current study, both filled delay conditions, which required the completion of a sustained attention task, produced an interference effect, relative to rest. It is possible that completion of these tasks generated sufficient interference—in the form of sensory and attention load—to disrupt consolidation-related neural processes including the automatic replay of encoded traces, which is found to occur predominantly during states of quiescence, including rest^18–20,44^. 

Finding further evidence that a restful, task-free delay is conducive to consolidation reinforces existing work^6–16^, but it is more intriguing that we observed no evidence of a trade-off between attention load and forgetting. Memory for encoded stimuli was comparable following the completion of sustained attention tasks that were low and high in their attention load. In fact, Bayesian analyses provided strong evidence in favour of the null, i.e., that delayed d’ scores were comparable following lower and higher attentional load variations of the SART. The two task variations were matched in sensory load: they used identical stimuli, the same number of trials, and the same trial timings. They only differed in the order of stimuli presented, with a fixed presentation in the SART-F and a random order of presentation in the SART-R. This methodological aspect is important given that sensory load may contribute to the induction of consolidation interference when completing an engaging task^17^. Our data support the expectation that the SART-R would be greater in attention load: participants made more commission errors (failed inhibition) and were slower to respond to trials in the SART-R than SART-F condition. Furthermore, performance levels in both SART variants, including error rates and response times, were in keeping with published work^42,45,46^, which suggests task validity in this online work, where experimental control cannot be guaranteed. It is, therefore, unlikely that issues in task design or participant engagement contributed to the lack of difference in delayed memory between the two task engagement delay conditions. Rather, our findings suggest that attention load does not determine consolidation interference on an incremental basis. 

Still, we cannot rule out the possibility that attention contributed to the observed effects in a broader sense. Specifically, it is possible that the level of sustained attention in both SART conditions was sufficient to meet a threshold to *induce* and (possibly more importantly) *maintain* a state of alertness, which was conducive to the encoding of new information but less so to the consolidation of recently encoded memories^4,5^. Thus, an incremental relationship between attention load and consolidation interference remains possible for tasks that place more casual and fluctuating demands on attention. This possibility aligns with the opportunistic consolidation hypothesis^3,4,32^, where even brief spontaneous microstates of quiescence (in the absence of sustained attention) are known to be conducive to consolidation^31–33^. This may account for conflict in current literature, for example, where no interference effect has been observed when using filled delays comprising n-back and visual matrices tasks^8,35^. This may be because such tasks did not place sufficient demands on attention to induce and maintain a state of alertness, thus enabling at least some spontaneous entry into quiescent states that could support consolidation. Further work is required to investigate these possibilities. 

Further to the above, it is worth noting that our findings conflict with some existing work^35^ suggesting that consolidation interference depends on the processing of rich sensory information with episodic properties, for example, photos of context-rich real-world scenes in a picture search task^6,9,11,17,23,25^ opposed to numeric stimuli in an n-back task or line drawings in a visual matrices task^8,35^. Findings are however mixed, where interfering effects of visual matrices have been reported in some cases^8^. Outcomes from the current study suggest that episodically rich stimuli are not required to induce an interference effect. Rather, our data indicate that an interference effect can be observed should a task involve sensory input and sustained attention. The exact contribution of sensory input and load and possible interactions with attention remain to be established. 

Finally, the findings from the current study speaks to the applied potential of minimising consolidation interference as a non-invasive intervention to promote memory retention in naturalistic settings. Such interventions could be especially advantageous to individuals with memory impairments (e.g., due to Alzheimer’s Disease) who have been shown to benefit strikingly from quiet rest, relative to controls^47–49^. Current evidence suggests that modest but significant rest effects in healthy younger and older controls often equate to ~ 10% superior retention following rest than task engagement^8,12,16,17,34^, whereas benefits in patient populations can reach upwards of 50%^26–28,50^ and are even observable after 7 days^26^. The outcomes reported here provide tentative but promising evidence that quiescent states in naturalistic settings are sufficient to positively affect memory retention. While the remote and self-report nature of some of our measures mean some caution is needed in interpreting our findings, observing a rest effect in memory through an online study is especially pertinent given that recent work has failed to observe effects of filled vs. unfilled delays in online research^47–49^. Further research is required on this specific aspect of awake consolidation, including exploration of the specific environmental and individual conditions that are conducive and detrimental to consolidation and the barriers to achieving a state of quiescence^47^. 

In conclusion, our data indicate that post-encoding task engagement not attentional load is detrimental to awake consolidation, relative to a state of minimal interference achieved through quiet rest. Further work is required to determine (i) whether the maintenance of sustained attention beyond a specific threshold contributed to our findings and (ii) the other factors that contribute to consolidation interference, including sensory load. Characterising consolidation can have implications for memory strategies and interventions in applied settings, especially for those with altered memory functioning who benefit most from states of minimal interference.

## Methods

### Ethics statement

This research was approved by the Faculty of Health and Life Sciences' Research Ethics Committee at Northumbria University (Ref: 3775). Informed online consent was acquired from all participants following an initial study briefing and procedures adhered to the appropriate ethical principles for research in humans.

### Subjects

An a priori sample size calculation was conducted using G*Power 3.1^51^. This calculation indicated that a minimum sample of 73 participants was required to detect a significant main effect of delay condition (three levels) in a repeated measures ANOVA when considering 80% power, an alpha level of 0.05, and a medium effect size (f = 0.25). Related laboratory findings have often demonstrated large effect sizes^6^ though findings are mixed for online work^47^; to this end, a more conservative medium effect size was used in the power calculation for the current study. The minimum sample required was exceeded through the recruitment of 120 young adults (women: n = 58, men: n = 57, non-binary: n = 3, agender: n = 1; gender fluid: n = 1; mean age = 28.26 years, SD = 4.25, age range: 18–35 years) as participants. These individuals were recruited between 21st June and 5th July 2023 through Prolific's participant panel and reimbursed at a rate of £10.47/hour. Data were accessed for analyses following completion of data collection, i.e., from 5th July 2023. Participants were not identifiable through Prolific or the information they provided during the study. Balanced sampling was used to ensure equal representation of self-identifying men and women. Inclusion criteria (applied through Prolific) comprised residing in the United Kingdom, fluency in English, no non-correctable hearing or visual impairments, and no known language disorders. There were no other exclusion criteria.

### Design

**Figure 4:**
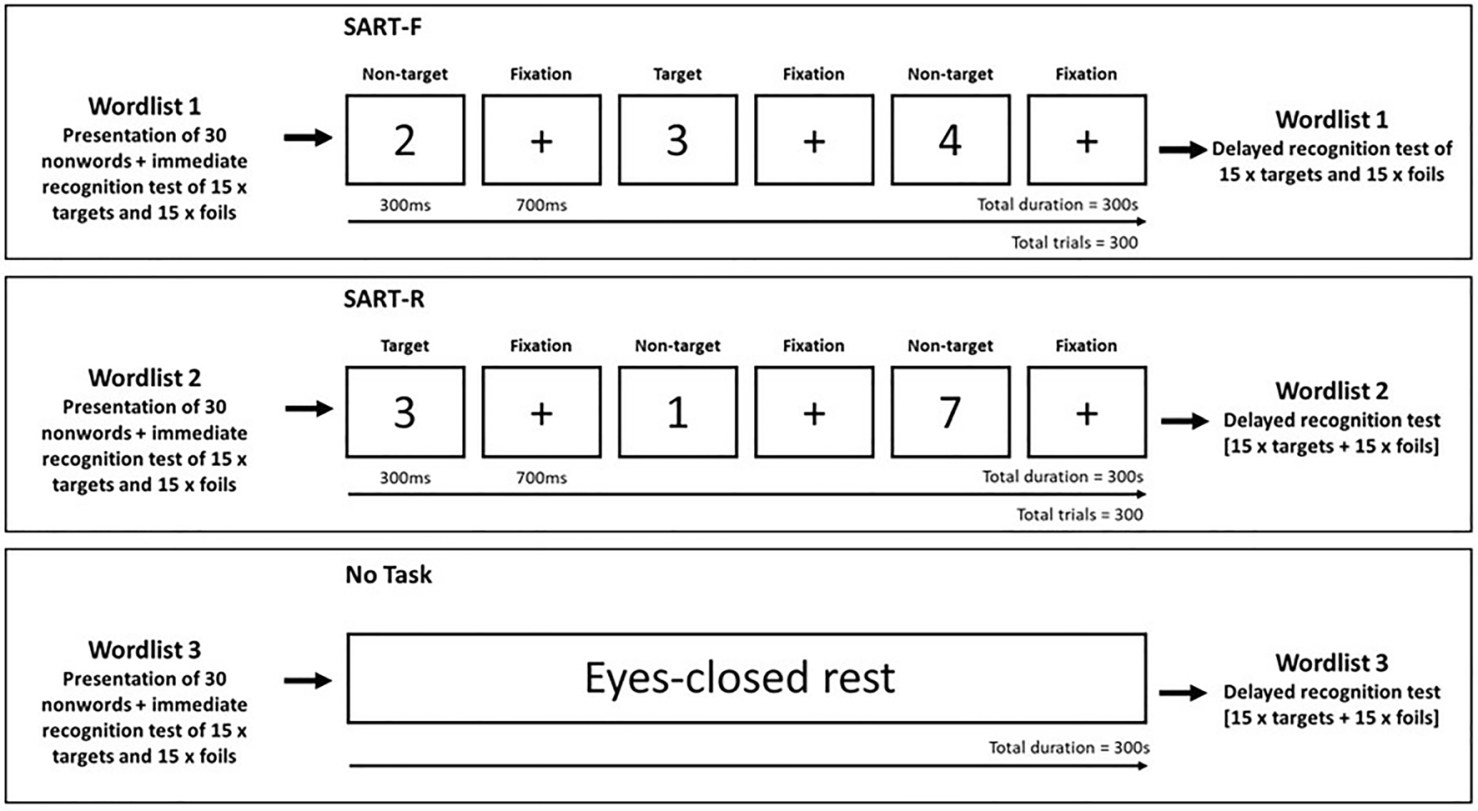
Experimental paradigm. Participants underwent three study phases, each comprising the encoding of one of three lists of 30 nonwords, an immediate recognition test for half of the nonwords, a 5-min delay condition, and a delayed recognition test for the remaining half of the nonwords. During encoding, each nonword was presented visually on the computer screen for 2000 ms and was followed by a 500 ms inter-stimulus crosshair (+). The 5-min delay between the immediate and delayed recognition tests comprised one of three delay conditions: (i) a sustained attention task with fixed order of numeric digits from 1 through to 9 (SART-F; low attention), (ii) a sustained attention task with random order of numeric digits from 1 through to 9 (SART-R; high attention), and (iii) no task condition, where participants were requested to sit quietly with their eyes closed for the duration of the delay. In both SART tasks, participants were required to respond to all digits with a keyboard (spacebar) response except for the number 3, where they were required to inhibit their response, i.e., not press the spacebar. In each recognition test, participants were presented with 15 nonwords from the initial set of stimuli (targets) along with 15 new nonwords (foils). Different foils were used in the immediate and delayed recognition tests. There was no limit on the time to respond during testing. Lists of nonwords and delay conditions were randomised fully across participants and gave 36 possible combinations.

We employed a repeated measures design with three within-subject delay conditions to examine the effect of post-encoding activities on the retention of nonwords. The experimental procedure was divided into three parts, which each comprised three phases: encoding, 5-min delay, and testing. Our experimental manipulation (rest vs. low attention vs. high attention; the attention tasks were based on Robertson et al., 1997) occurred during the delay phase (see Fig. 4). Participants completed the study on their personal laptop or PC device. The study was completed in a single session lasting approximately 30 min.

## Materials

Our experimental paradigm used a variation of an awake consolidation paradigm that has been shown to detect rest-related effects in memory^6,10,11,16,21,34^. The computerised task used to administer our experimental paradigm was developed using PsychoPy3 (version 2021.1.4) via a Python-coded script and delivered online via pavlovia.org. The experimental task is available on the project OSF site at osf.io/s8fj7. The experimental task was accompanied by pre- and post-study questionnaires that were delivered through Qualtrics. 

### Procedure

Participants registered their interest in the study through Prolific and were redirected to a pre-study Qualtrics comprising an information sheet and consent form. If, after reading the information sheet, the participant provided their informed consent, they were first asked to provide demographic information, including their age, gender, and level of educational attainment. 

Participants then completed our experimental procedure, which was divided into three phases. Each phase probed immediate and delayed memory for a list of nonwords (e.g., *remaven*) either side of a 5-min delay condition and comprised of (a) an encoding phase, (b) an immediate recognition test, (c) a 5-min delay condition, and (d) a delayed recognition test. Within each encoding phase, participants were presented 30 nonwords in a randomised order. Each nonword was presented visually in the centre of the computer screen for 1000 ms and was followed by a 1000 ms inter-stimulus interval, which comprised the presentation of a small crosshair (+) in the centre of the screen. Participants were asked to attend to the words and try to remember them as best as possible as their memory for the nonwords would be tested immediately after the presentation. Three different lists of nonwords were used across the three phases of the experiment. The order of the three lists across the three study phases was randomised. Nonwords were generated through the Auditory English Lexicon Project (AELP) database^52^. All nonwords were three syllables, comprised 7–12 letters, and were based on common nouns, for example, *cartilage* became *cartolale* and *inhaler* became *inholen*. In each list, word initials were distributed across the alphabet.

Following the encoding phase, participants’ memory for the presented nonwords was probed. This was achieved through a recognition test, where they were required to note whether an item was an old item (target) that appeared in the recent presentation or a new item (foil) that did not. To avoid potential confounds associated with the retrieval of encoded materials influencing consolidation positively^43^, only half of the encoded items were probed in the immediate recognition test as targets (total = 15). The remaining 15 items were probed in the delayed recognition test (see later). The 15 target items were accompanied by 15 foils. Participants responded via the computer keyboard, where ‘z’ = old item and ‘m’ = new item. As during encoding, test stimuli were presented visually in the centre of the screen. The order of test items was randomised to reduce the possibility of order presentation effects and there was no limit on the time to respond to probe trials.

Participants then experienced one of three 5-min delay conditions, where they were asked to either rest quietly or complete a sustained attention to response task (SART)^42^. Two variations were used: the SART-F used a fixed order of stimuli presentation (digits 1 to 9 presented in a sequential order, i.e., 1–2-3–4-5–6-7–8–9–1–2…), and the SART-R used a random order of stimuli presentation (digits 1 to 9 presented in a random order, e.g., 4–5–7–6–3–1–2–8–9–2–6…). In both variations, participants responded to all items except the digit 3, where response inhibition was required. Omission errors refer to a missed response, i.e., where no response was provided to digits 1–2 and 4–9. Commission errors refer to failed inhibition, i.e., where a response was incorrectly made to the digit 3. Because of this structure, the SART-R is typically considered to place greater demand on attentional resources. Figure 3 shows participants’ performance in the SART-F and SART-R conditions. In the rest delay condition, participants were asked to sit quietly and rest with their eyes closed for the duration of the condition. This was in attempt to minimise the experiencing of rich visual and/or audible sensory cues which could disrupt consolidation. Throughout the duration of the rest delay, participants were presented with a black screen, which was overlaid with a small white cross in the centre of the screen to demonstrate to the participant that the experimental task remained active. Five seconds before the end of the rest delay, participants were presented a 1000 ms ‘A’ tone to indicate that they had reached the end of the rest period. Instructions for the rest and SART delay conditions were presented on screen immediately following the end of the incidental encoding phase and immediately prior to the allocated 5-min delay condition.

Following each delay phase, participants’ memory for the nonwords was again probed through a further two-force choice recognition test. The format of this test was identical to the immediate recognition test except a different set of 15 targets from the earlier encoding phase and 15 new foil items were used. 

At the end of the testing phase, participants completed a self-report post-experimental questionnaire to assess their mental activities pertaining to encoded items during the three delay conditions. To this end, for each delay, participants were asked whether they engaged in any strategies to help them remember the encoded nonwords and whether they experienced any challenges in completing the delay condition. Further to this, using an existing scale from related research^12^, participants were asked to rate the regularity that they (i) thought about, (ii) imagined, and (iii) remembered the recently presented nonword stimuli during a delay condition. This was probed for each delay condition, where participants were required to provide a rating on a 5-point Likert scale, where 1 = not at all and 5 = constantly.

### Scoring

Performance in our memory test was examined in keeping with signal detection theory^53^. For the immediate and delayed recognition test of each delay condition, we extracted the total number of correct responses to old (target) items and new (foil) items. From these values, hit rate (number of correct “old” responses to targets / total number of targets) and false alarm rate (number of incorrect “old” responses to foils / total number of foils) scores were computed. In instances where a rate of 0 or 1 were found, these rates were corrected. Specifically, in keeping with recommended signal detection theory corrections^54,55^, a rate of 0 was replaced with 0.5/n and a rate of 1 was replaced with (n-0.5)/n. These scores were then used to compute d prime (d’) scores using the standard formula: z(hit rate) − z(false alarm rate), which was done to record how well old items were discriminated from new items. Finally, we extracted the time (ms) that it took participants to respond during the immediate and delayed recognition tests of each delay condition to check for potential differences in retrieval.

### Statistical analyses

Inferential and Bayesian analyses were performed using SPSS Statistics 28 (copyright IBM Corp., NY, USA), with the alpha level set to 0.05 for the latter. Descriptive statistics were computed for participant demographics, performance in our experimental procedure, post-experimental reports of mental activities during our experimental procedure, and scale-based trait measures. Repeated measures analyses of variance (ANOVAs) and follow-up paired t-tests were conducted to examine possible differences between our post-encoding delay conditions (rest vs. low attention vs. high attention) across memory scores (e.g., d’ values). Bonferroni-corrected alpha levels were applied (P = 0.05/# comparisons) to correct for multiple within-family comparisons. Bayesian paired sample t-tests were used as follow-up analyses to examine whether, in cases of non-significant findings between delay conditions, there was evidence for or against the null hypothesis. As we did not have previous data to base prior assumptions, the default Cauchy(0,1) prior for effect size (r = 0.707) was used^56^.

## Author contributions 

Both authors conceptualised the study. M.C. developed the computerised PsychoPy task. Both authors performed data analyses, contributed to writing the paper, and approved the paper before submission.

## Data Availability

Task (e.g., PsychoPy) files, stimuli, and the data that support the findings of this study are freely available on the project OSF site at osf.io/s8fj7.
